# Genistein attenuates retinal inflammation associated with diabetes by targeting of microglial activation

**Published:** 2010-10-08

**Authors:** Ahmed S. Ibrahim, Mamdouh M. El-Shishtawy, Alejandro Peña, Gregory I. Liou

**Affiliations:** 1 Department of Ophthalmology, Medical College of Georgia, Augusta, GA; 2 Department of Medicine, Medical College of Georgia, Augusta, GA; 3 Department of Biochemistry, Faculty of Pharmacy, Mansoura University, Mansoura, Egypt

## Abstract

**Purpose:**

Diabetic retinopathy (DR) is associated with microglial activation and increased levels of inflammatory cytokines. Genistein, a tyrosine kinase inhibitor, has been shown to possess anti-inflammatory potential that so far untested in animal models of diabetes. The aims of this study are to evaluate the efficacy of genistein for alleviation of diabetes-induced retinal inflammation and also to gain insight into the molecular mechanisms involved therein by analyzing the effect of genistein on concomitant microglia activation in the diabetic retina and in isolated cells.

**Methods:**

Streptozotocin (STZ)-induced diabetic Sprague Dawley rats were used. After diabetes was established for two weeks a single intravitreal injection of genistein or vehicle was performed. Forty-eight hours later, rats were killed, their retinal and vitreal samples were processed for Quantitative Real Time-PCR (qRT–PCR) and Enzyme-linked immunosorbent assay (ELISA) analyses, respectively. For the in vitro study, isolated microglial cells from retinas of newborn rats were used.

**Results:**

mRNA as well as protein levels for tumor necrosis factor α (TNF-α), a robust marker of inflammation, were increased in the retina early in the course of diabetes. Moreover, diabetes resulted in elevation of ionized calcium binding adaptor molecule-1 (*Iba1*) mRNA, known to be upregulated in activated microglia. These effects of diabetes in retina were all reduced by intervention treatment with genistein. Using an in vitro bioassay, we demonstrated the release of TNF-α from microglia activated by glycated albumin, a risk factor for diabetic disorders. This inflammatory signal involves the activation of tyrosine kinase and its subsequent events, ERK and P38 MAPKs. Genistein represses the release of TNF-α and significantly inhibits ERK and P38 phosphorylation in activated microglial cells by acting as a tyrosine kinase inhibitor.

**Conclusions:**

These findings show genistein to be effective in dampening diabetes-induced retinal inflammation by interfering with inflammatory signaling (ERK and P38 MAPKs) that occurs in activated microglia. This beneficial effect of genistein may represent a new intervention therapy to modulate early pathological pathways long before the occurrence of vision loss among diabetics.

## Introduction

Diabetes is a global health problem and its prevalence is set to increase up to 366 million worldwide by the year 2030 [1]. Persistent hyperglycemia in diabetic patients despite appropriate therapeutic measures leads to several complications including retinopathy, nephropathy, and neuropathy. Diabetic retinopathy (DR) is the most prevalent microvascular complication affecting approximately 50% of diabetic patients within 15 years after onset of the disease [2]. Complications from the condition may include gradual loss of sharpness of vision, and at their most extreme, total loss. Laser treatment which has been a standard therapy for the past 25 years, does not prevent DR nor is it used to treat mild or moderate DR, rather it is used to treat only serious, vision threatening DR. Therefore, there is a great need for the development of new and effective therapies for treating DR early before it causes irreparable damage to the retina.

The etiology of diabetic retinopathy is complex and multi-factorial. During the past decade, it has been suggested that inflammation, induced by hyperglycemia or glycated proteins, is a central contributing factor in pathogenesis of diabetic retinopathy [3]. Clinical studies have shown elevated levels of pro-inflammatory cytokines in the vitreous fluid of patients with diabetic retinopathy, which are related with the activity and progression of retinal injury [4]. Experimental animal investigations have shown that mRNA expression for TNF-α, a robust marker of inflammation, is increased in the retina early in the course of diabetes, and moreover, inhibition of TNF-α has demonstrated beneficial effects in the prevention of early diabetic retinopathy [5].

Cellular sources of these inflammatory mediators include infiltrated leukocytes and the pre-stationed glial-immune cells known as-microglia. The importance of retinal microglia as responders to hyperglycemic stress has recently been emphasized in experimental diabetes. In vitro cellular models, activation of microglia cells with glycated albumin or high glucose has been used as a model to simulate inflammation during diabetes [6]. In a diabetic animal model, streptozotocin (STZ)-induced diabetic rats have been shown to develop retinal microglia activation leading to the release of soluble cytotoxins that contribute to neuronal and vascular cell death and ultimately the progression of DR [7]. As such, pharmacologic regulation of microglial activity is therefore a rational approach to modulate early pathological pathways associated with DR long before the occurrence of vision loss among diabetics.

Genistein, an isoflavonoid, is a naturally occurring tyrosine kinase inhibitor that has recently attracted considerable attention, as it has a wide therapeutic index [8] and it is effective in several animal models in which microglia and inflammation have been implicated. Genistein protects dopaminergic neurons from lipopolysaccharide-induced injury by inhibiting microglial activation [9]. It also has been shown that genistein reduces pro-inflammatory factor-induced vascular endothelial barrier dysfunction and inhibits leukocyte-endothelium interaction, the major events in the pathogenesis of atherosclerosis [10]. Moreover, it has proven that genistein protects neurons from transient global cerebral ischemia/reperfusion injury in rat hippocampus [11]. However, the effects of genistein on retinal inflammation and microglia activation during diabetes have not been reported. The present study evaluates the ability of intravitreal genistein to attenuate diabetes-induced retinal inflammation. Furthermore, this study pursues to gain insight into the mechanistic basis behind this effect. Because genistein has an attractive safety profile and appreciable anti-inflammatory activity, it may represent a potential therapeutic agent for managing retinal complications during diabetes.

## Methods

### Materials

Genistein was purchased from LC laboratory (Woburn, MA). Antibodies against glial fibrillar acidic protein (GFAP), phospho-tyrosine, phospho-ERK (pERK), ERK, phospho-P38 (pP38) and P38 were purchased from Cell Signaling (Beverly, MA). Anti-Iba1 was purchased from Wako (Richmond, VA). Anti-glycated albumin antibody (A717) was purchased from Exocell (Philadelphia, PA). P38 inhibitor, SB203580, was purchased from Calbiochem (Gibbstown, NJ). β-actin antibody, ERK inhibitor (U0126), glycated and nonglycated albumin were purchased from Sigma (St. Louis, MO). Glycated albumin contained 3 mol of fructoselysine per mol albumin. The absence of advanced glycation end products (AGE) in the glycated albumin was assessed by measuring of AGE-related fluorescence at excitation maximum of 370 nm and emission maximum of 440 nm as described previously [12]. Endotoxin was not detectable in the glycated or nonglycated albumin by the use of Limulus Amebocyte Lysate test kit.

### Animal preparation and experimental design

All procedures with animals were performed in accordance with the Public Health Service Guide for the Care and Use of Laboratory Animals (Department of Health, Education, and Welfare publication, NIH 80–23), Medical College of Georgia guidelines and The Association for Research in Vision and Ophthalmology Statement for the Use of Animals in Ophthalmic and Vision Research. Diabetes was induced for 2-weeks in male Sprague-Dawley (SD) rats (bodyweight, 200 g) by intravenous injection of STZ (60 mg/kg) in sodium citrate. Detection of glucose in the urine and blood glucose levels >250 mg/dl were the markers to indicate diabetic status. The diabetic rats were randomly divided into two subgroups: genistein-treated and vehicle-treated subgroups.

### Intravitreal injection

This procedure was essentially the same as previously described [13]. To avoid uncontrolled intraocular pressure increase, the volume of intravitreal injections was limited to 2 µl. Genistein was dissolved in dimethyl sulfoxide (DMSO) and a working solution of 25x was prepared by diluting 1.25 µl of stock solution (100 mM) to 100 µl with phosphate-buffered saline (PBS), assuming the vitreous volume of rat eye is 50 µl [14]. Then by injecting 2 μl of this working solution, 50 µM vitreal concentration of genistein was obtained. The vitreal concentration of DMSO (DMSO) was 0.05%. To ensure the proper delivery and even distribution of the intravitreally injected compounds, all solutions for intravitreal injection contained 5 µg/ml of Fast Green FCF (Sigma, St. Louis, MO). The volume of the injected solution apparently did not cause significant pressure-induced retinal damage, because PBS-injected control eyes showed normal retinal morphology with no apparent apoptosis within 7 days. Intravitreal injections were performed in 2-weeks diabetic rats. 48 h later, rats were killed, and their retinal as well as vitreal samples were collected separately. The retinas were removed, snap frozen in liquid nitrogen, stored at −80 °C, and analyzed by Quantitative Real Time-PCR (qRT–PCR) or western blot, while the vitreal samples were centrifuged and used for the measurement of TNF-α release by ELISA. (n=6 per group).

### Primary retinal microglia culture

Microglial cells were isolated from retinas of newborn Sprague Dawley (SD) rats according to a previous procedure [15], with minor Modifications. Briefly, retinas were collected into 0.01 M PBS and digested with 0.125% trypsin for 3–5 min before mixing with Dulbecco’s Modified Eagle Medium (DMEM)/F12 containing 10% fetal bovine serum (FBS) and 1% penicillin/streptomycin. Retina pieces were then filtered through a mesh (100 µm), collected by centrifugation, resuspended in culture medium and plated onto T150 cell culture flasks (Corning, NY) at a density of 2×10^5^ cells/cm^2^. After 2 weeks, microglial cells were harvested by shaking the flasks at 100 rpm for 1 h. Immunocytochemical studies showed that more than 95% cultured cells stained positively for Iba1. Almost none of these cells showed positive staining for GFAP, indicating that majority of the isolated cells were microglia and were not contaminated with astrocytes or Müller cells (data not shown). For microglia activation, glycated albumin was added to each well in Cellgro Complete media for indicated time. Non-glycated albumin was used as a control. The final concentrations of drugs were: genistein, (10–100 µM); U0126, 10 µM; SB 203580, 10 µM. Cell viability was determined by counting the number of trypan blue–excluding cells under an inverted microscope, using a hemocytometer. Cells were homogenized for western blot analysis. Culture media were used for TNF-α release determination by ELISA.

### Quantitative Real Time-PCR

Total RNA was isolated from rat retina using Promega SV Total RNA Isolation System. Subsequently, cDNAs were generated from 1 μg of total RNA using High-Capacity cDNA Reverse Transcription Kit (Applied Biosystems). The resulting cDNA was subjected to a 40 cycle PCR amplification using manufacturer’s TaqMan Universal PCR Master Mix protocol. Quantification of *Iba-1*, *TNF-α*, and Glyceraldehyde-3-phosphate dehydrogenase (*GAPDH*) transcripts were performed by relative quantitative real-time RT–PCR with TaqMan® Probe-based Assays and Applied Biosystems 7300 Sequence Detection system. The ready-made primer and probe sets were ordered from Applied Biosystems (Catalog #: Iba-1: Rn01525935_m1; TNF-α: Rn99999017_m1; GAPDH: Rn01775763_g1). Three replicates were run for each gene for each sample in a 96-well plate. *GAPDH* was used as the endogenous reference gene as it does not exhibit significant expression changes between groups of samples (data not shown). The relative quantitation method (ΔΔCt) was used, with the ratio of target mRNA, normalized respect to GAPDH mRNA and relative to a calibrator sample. PBS-normal, non-diabetic, retinas were used as calibrators.

### Enzyme-linked immunosorbent assay (ELISA) for TNF-α in vitreous or culture media

TNF-α levels in the samples were estimated with ELISA (R&D) per the manufacturer’s instructions. Briefly, standards and samples were bound by the immobilized antibody, and an enzyme-linked polyclonal antibody specific for the cytokine was added to the wells followed by a substrate solution yielding a colored product. The intensity of the color was measured at 450 nm. The sample levels were calculated from a standard curve and were corrected for protein concentration.

### Immunolocalization of TNF-α and retinal glial cells

Retinal frozen sections (10 µm) were prepared for immunofluorescence and incubated with the microglial cell-specific marker anti-Iba-1 together with anti-TNF-α (Santa Cruz). This was followed by incubation with Texas red- and Oregon green-labeled secondary antibodies (Molecular Probes). The same procedure was applied to astrocytes/Mueller cell marker anti-GFAP, together with anti-TNF-α followed by Texas red- and Oregon green-labeled secondary antibodies. Slides were examined by confocal microscopy (LSM 510, Carl Zeiss). Specificity of the reaction was confirmed by omitting the primary antibody. Images were collected from five sections per rat of at least three rats in each group.

### Western blot analysis

Retina and cell homogenates was subjected to western blot analysis according to a previous procedure [15]. Antibodies for β-actin, glycated albumin, phospho-tyrosine, phospho-ERK and ERK, phospho-P38 and P38 were detected with a horseradish peroxidase-conjugated antibody and enhanced chemiluminescence detection system (Amersham BioSciences). Intensity of immunoreactivity was measured by densitometry.

### Data analysis

The results were expressed as mean ± SD. Differences among experimental groups were evaluated by ANOVA, and the significance of differences between groups was assessed by the posthoc test (Fisher’s PLSD) when indicated. Significance was defined as p<0.05.

## Results

### Genistein attenuates retinal inflammation during diabetes

**Figure 1:**
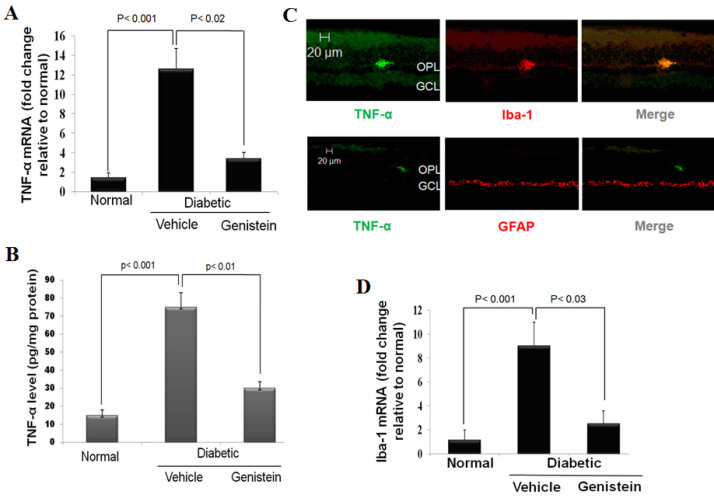
Effect of intravitreally injected genistein on Streptozotocin (STZ)-induced retinal inflammation and microglial activation. 2-weeks diabetic rats were treated with genistein (50 µM) or vehicle (0.05% DMSO) intraocularly. 48 h later rats were killed and their retinal and vitreal samples were processed for Quantitative Real Time-PCR (qRT–PCR) and enzyme-linked immunosorbent assay (ELISA) analyses, respectively, (n=6). **A**: qRT–PCR analysis for *TNF-α* expression in diabetic rat retinas after 48 h treatment with genistein or vehicle intraocularly. The level of gene expression was presented as the mean fold change±SD relative to normal non-diabetic rats. **B**: ELISA analysis of vitreal TNF-α release in vitreous and expressed as absolute value±SD **C**: Representative images show the colocalization of TNF-α with glial markers in the retina of 2-weeks diabetic rats. Iba-1 (red), a marker of activated microglia; glial fibrillary acidic protein (GFAP; red), a marker of astrocytes or activated Müller cells. Yellow displayed from merged red and green. Scale bar represents 20 μm. Abbreviations:OPL indicates outer plexiform layer; GCL indicates ganglion cell layer; (magnification, 200×). **D**: Effect of intravitreally injected genistein on microglial activation assayed by *Iba1* mRNA expression using qRT–PCR. The level of gene expression was presented as the mean fold change±SD relative to normal non-diabetic rats.

Given the inflammatory nature of early diabetic retinopathy [16] and the fact that the tyrosine kinase signaling cascade plays a pivotal role in initiating activation of various inflammatory cells [17], we hypothesized that genistein, a tyrosine kinase inhibitor, may also be effective in attenuation of diabetes-induced retinal inflammation. To test this, the effect of intravitreal genistein on retinal *TNF-α* mRNA expression as well as protein levels were determined. In this study, 2-weeks diabetic rats were treated with genistein or DMSO intraocularly. After 48 h, *TNF-α* mRNA expression was determined by Quantitative Real Time-PCR and vitreal TNF-α was determined by ELISA. As shown in Figure 1A,B, intravitreal injection of genistein resulted in significant inhibition of STZ-induced *TNF-α* expression and release. Furthermore, the results of these analyses confirmed previous reports that inflammation is an early and important component of diabetic retinopathy [7,18].

### Genistein reduces microglial activation in the diabetic retina

After having shown that genistein exhibits profound reduction in both *TNF-α* expression and release, we next sought to explore a potential mechanism by which genistein regulates inflammation in diabetic retinopathy. First, we performed additional studies to identify the main source of TNF-α in the diabetic retina. Because glial cells (microglia and macroglia) are the most potential candidates for inflammatory cytokine production, we used double labeling with specific glial markers (Iba-1 for activated microglia and GFAP for astrocytes or Müller cells) to determine the cellular source of TNF-α production. Through immunofluorescence, TNF-α was colocalized with Iba-1 but not with GFAP, indicating that microglia are the main cell type producing TNF-α; at least during the 2-weeks of diabetes (Figure 1C**)**. Next, we aimed to determine whether the curative effect of genistein was mediated by targeting of microglial activity. To test this, the effect of intravitreal genistein on microglial activation was determined by measuring *Iba1* expression, which is upregulated in activated microglia. To monitor microglial activation, the level of *Iba1* mRNA was quantified in retinas after 48 h of genistein or vehicle treatment. As assessed by Quantitative Real Time-PCR, whole retina *Iba1* mRNA in genistein-treated rats was approximately 75% lower on average than in vehicle-treated diabetic rats (Figure 1D**).** Together, these findings suggest that genistein reduces STZ-induced retinal inflammation by dampening microglial cell activation.

### Genistein, a tyrosine kinase inhibitor, mitigates TNF-α release in stimulated retinal microglial cells

**Figure 2:**
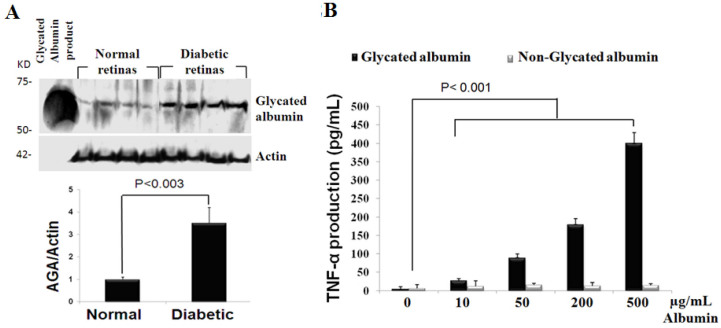
Accumulation of glycated albumin in diabetic retina and its inflammatory potential for microglia cells. **A**: Glycated albumin expression in normal, 2-weeks diabetic rats, and 12.5 ng of glycated albumin product (Sigma), analyzed by western blot using mouse monoclonal antibody A717. The mean ratio±SD of the intensity of glycated albumin versus actin was indicated below each group. The ratio of normal, non diabetic rats was taken as 1.0. **B**: Dose-dependent release of tumor necrosis factor α (TNF-α) in glycated albumin-treated microglial cells. Microglial cells were stimulated with 10, 50, 200, 500 µg/ml glycated albumin for 4 h. TNF-α levels assayed by enzyme-linked immunosorbent assay (ELISA) in culture supernatant were compared with corresponding dosage of non-glycated albumin-treated cells and are expressed as means±SD for three independent experiments.

From aforementioned evidence that highlighted the anti-inflammatory potential of genistein by targeting of microglial activity in STZ-injected rats, a cultured retinal microglia model was developed. This cultured model is advantageous because it offers a more detailed characterization of genistein’s anti-inflammatory actions and also helps elucidate the molecular mechanism responsible for this effect. In this model, we determined the ability of genistein to affect TNF-α release from retinal microglia in response to glycated albumin, a risk factor for diabetic disorders. Glycated albumin was found to be increased in the retina of 2-weeks diabetic rats (Figure 2A) and has been shown to elicit the inflammatory response in isolated microglia cells. This inflammatory response was characterized by a dose-dependent TNF-α production after 4 h incubation with increasing amounts of glycated albumin (Figure 2B).

**Figure 3:**
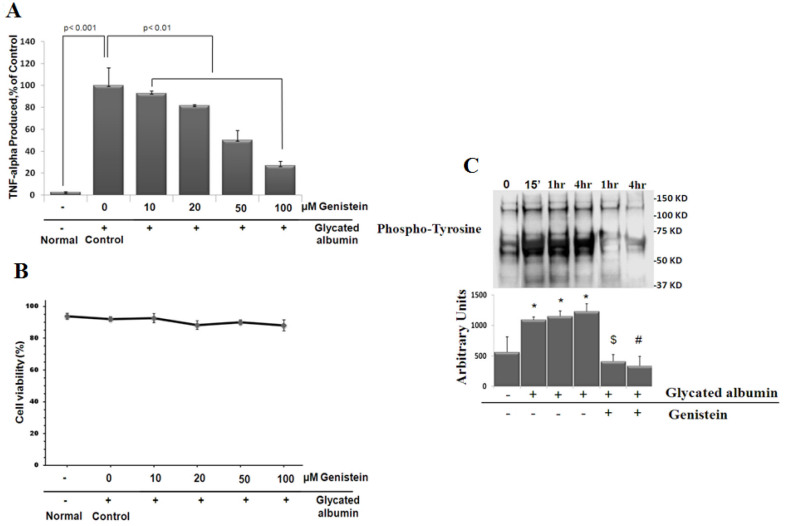
As a tyrosine kinase inhibitor, genistein mitigates tumor necrosis factor α (TNF-α) release in stimulated retinal microglia cells. **A**: Genistein’s dose-dependent inhibition of TNF-α release from activated microglia. Cells were treated with (500 μg/ml) glycated albumin for 4 h in the presence of indicated concentrations of genistein. TNF-α released was analyzed by enzyme-linked immunosorbent assay (ELISA). Values represent the means percentage±SD of TNF-α release compared with that of glycated albumin-treated in presence of vehicle for three experiments. **B**: Genistein also had no effect on cell viability, as determined by trypan blue exclusion test. **C**: Time-dependent, glycated albumin-induced tyrosine phosphorylation in microglial cells. Cells were treated with (500 μg/ml) glycated albumin in the presence or absence of 100 µM genistein for the indicated time. Phosphorylated tyrosine was determined by Western analysis. Intensities of phosphorylated tyrosine for each time points were compared with the control (time 0). Data shown is the mean±SD of three experiments. * p<0.001 compared with 0 time; $ p<0.001 compared with non-genistein treated, 1 h; # p<0.001 compared with non-genistein treated, 4 h.

Of note, the increment of TNF-α level is significantly measureable at 4 h after glycated albumin treatment and continued for 24 h (Ibrahim et al. submitted). Moreover, this early time point was chosen in this study to minimize the positive feed-back effect of TNF-α [19]. The concentration of glycated albumin (500 µg/ml) chosen in our study is close to what has been used previously by several investigators to study other glycated albumin-mediated responses such as stimulation of glomerular endothelial and mesangial cell expression of collagen type IV [20] and represents those found in clinical specimens. In non diabetic individuals, ~1% of serum albumin is in the glycated form, which is equivalent to concentrations of 300–400 µg/ml of glycated albumin. The concentration of glycated albumin is increased one-and-a-half- to threefold in diabetic subjects, according to recent glycemic status [21]. To assess the ability of genistein to reduce TNF-α release, microglial cells were pretreated with indicated concentrations of genistein for 1/2 h then stimulated with glycated albumin for 4 h. The supernatants were collected and assayed for TNF-α by ELISA. As shown in Figure 3A, genistein inhibited glycated albumin-mediated TNF-α release in a dose-dependent manner. To ensure that this effect was not caused by nonspecific cytotoxicity of genistein, we assessed cell viability in microglial cells 4 h after an exposure to genistein, using the trypan blue exclusion test. As shown in Figure 3B, genistein did not affect cell viability (88%–94% vital cells), which indicates that the decrease in TNF-α release was indeed consecutive to genistein’s anti-inflammatory effect but not to cell death.

To verify that the inhibitory effect of genistein on TNF-α release from activated microglia was mediated by protein tyrosine kinase inhibition, we first tested the potential of glycated albumin to induce tyrosine kinase activation. To address this point, microglial cells were treated with glycated albumin then at 1/4, 1, and 4 h later cultures were rinsed with PBS and the cells were lysed. Cell lysates were electrophoresed on SDS–PAGE gels, electroblotted onto nitrocellulose membranes, and probed with antibody to phosphotyrosine. Tyrosine phosphorylation of many proteins, determined by western blot, occurred at 15 min and continued over the 4-h experimental period **(**Figure 3C**)**. This result indicates that tyrosine phosphorylation is among the molecular events induced in microglia after glycated albumin treatment. Second, we examined the effects of genistein on glycated albumin-induced protein tyrosine phosphorylation in retinal microglia. Genistein was added 1/2 h before treatment with glycated albumin for 1 and 4 h and the tyrosine phosphorylation profile was determined. Glycated albumin-induced increases in the level of cellular phosphotyrosine were suppressed by pretreatment with genistein **(**Figure 3C**)**. This result clearly demonstrates the tyrosine kinase inhibitory effect of genistein. Moreover, the inhibitory effect of genistein on TNF-α release demonstrates that tyrosine phosphorylation is an early and causative event in glycated albumin-induced TNF-α release from retinal microglial cells.

### Genistein mediates anti-inflammatory effect by inhibiting glycated albumin-dependent phosphorylation of ERK and P38 in retinal microglial cells

**Figure 4:**
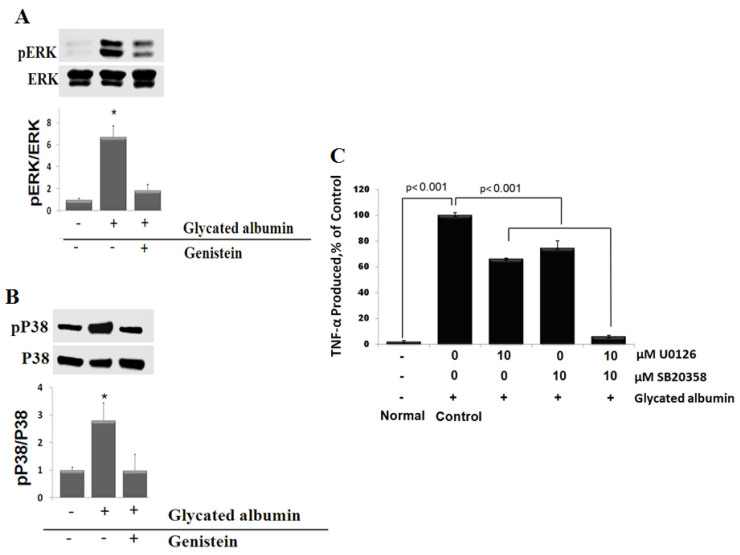
Genistein inhibits MAPK activation in stimulated microglial cells. **A, B**: Cells were treated with vehicle or 100 µM of genistein 30 min before (500 μg/ml) glycated albumin treatment for 4 h. Phospho-ERK, total ERK, Phospho-P38, and total P38 MAPK were determined. Each glycated albumin-treated samples with or without genistein were compared with the untreated sample, set as 1.0. Data shown is the mean±SD of three experiments. * p<0.001. **C**: Contribution of ERK and P38 MAPKs to tumor necrosis factor α (TNF-α) release in response to glycated albumin. Cells were treated with U0126 (10 µM) or with SB203580 (10 µM) either alone or in combination for 30 min. Cells were then treated with glycated albumin (500 μg/ml) for 4 h. TNF-α levels were compared to the vehicle-treated control. Data shown is the mean±SD of three experiments.

In light of genistein’s anti-inflammatory effect, interest in its mechanisms of action has expanded to include several protein kinase pathways. The mitogen-activated protein kinase superfamily is composed of several signaling pathways including extracellular signal-regulated kinase (ERK), c-jun NH2-terminal kinases (JNKs) and p38 MAPKs. The ERK and p38 MAPKs pathways are the primary signaling pathways that are activated in microglia treated with glycated albumin (Ibrahim et al., submitted). Whether or not these kinases are modulated by genistein was also examined. Microglial cells were pretreated with 100 µM genistein for 30 min and then stimulated with glycated albumin for 4 h; phosphorylation of ERK and P38 were then measured. Genistein inhibited most of the ERK phosphorylation induced by glycated albumin **(**Figure 4A**)**. In addition, genistein significantly reduced glycated albumin-stimulated P38 phosphorylation **(**Figure 4B**)**. Further, we determined ERK and P38 contribution to TNF-α release. Microglial cells were pre-treated with U0126 and SB203580 individually or in combination for 30 min and treated with glycated albumin for 4 h. The combination of ERK and P38 inhibitors resulted in almost totally inhibition of TNF-α release (Figure 4C). Together, these results suggest that genistein inhibits glycated albumin-induced expression of TNF-α through inhibition of ERK and P38 MAPKs. At the same time, these results indicate that phosphorylated tyrosines are common signaling molecules that are located upstream of both ERK and P38 pathways-mediating TNF-α release in glycated albumin-treated retinal microglial cells.

## Discussion

Recent biochemical and neurobiological studies have shown that retinal glial cell dysfunction and signs of inflammatory reactions [22], including TNF-α release, are relatively early events that occur in response to diabetes before vascular dysfunction involving acellular capillary formation and neovascularization [23]. Moreover, TNF-α has been shown to recruit leukocytes, cause vascular breakdown and promote neuronal injury at high levels. Thus, treatments targeting early features of diabetic retinopathy would provide long-term vascular benefits [16]. The in vitro and in vivo bioassay data in this study demonstrated the ability of genistein to counteract retinal inflammation during diabetes by dampening of microglia activation and TNF-α-release. Genistein has a wide therapeutic index with very low toxicity [8] and its efficacy has been demonstrated in different animal models of inflammatory diseases.

In relation to diabetes, genistein has shown a plethora of beneficial effects in experimental animals. Chronic treatment with genistein improved the functional changes in aortic vascular reactivity and inhibited retinal vascular leakage observed in diabetic rats [24,25]. Recently genistein has been reported for its potential hypoglycemic activity following chronic systemic administration. This anti-diabetic effect of genistein is mediated via preservation of insulin positive β-cells and restoration of the glucose metabolic enzyme activities independently from its tyrosine kinase activity [26]. Because hyperglycemia is known to be responsible for the functional abnormalities associated with diabetes, any beneficial effects of genistein might be secondary to its action on insulin secretion or hyperglycemia. However, the demonstration that genistein reduces the expression of proinflammatory cytokines in other diseases has raised the possibility of anti-inflammatory properties beyond its role as an anti-diabetic agent. In the context of diabetes, these two effects of genistein cannot be separated. In this study, we put forward a new concept pertaining to the therapeutic effect of genistein in treating diabetic retinal inflammation independently of its anti-diabetic property. To distinguish the possible retinal anti-inflammatory effects of genistein from its activity as an insulin releaser, we examined the local ocular effect of genistein in STZ-induced diabetic animals. The intraocular structures of the eye are isolated from the systemic circulation by both the inner and outer blood–retinal barriers, these barriers permit local delivery of active drug products directly while minimizing systemic absorption and side effects. Therefore, any anti-inflammatory effects of the drug were not secondary to its action on insulin secretion or hyperglycemia.

Intravitreal injection of genistein, in the present work, was found to cause a significant inhibition of *TNF-α* mRNA as well as protein levels in diabetic retina, demonstrating the curative effect of genistein on inflammation associated with STZ-diabetic model. Moreover, systemic adverse side effects that might be associated with genistein therapy have been addressed by using intravitreal injection for the drug. A 50 µM concentration (0.675 µg) of genistein was used for the intravitreal injection in this study. This concentration is well below the safety range of intravitreal genistein. At a 500-micromole concentration (0.135 g), genistein did not show any toxic effects, either on histological or electrophysiological examination of the retina [27]. Genistein also prevented retinal microglia from upregulating *Iba1* mRNA, supporting the hypothesis that genistein reduces the retinal inflammation and inflammatory cytokines expression through attenuation of microglia activation.

Following this further, we used primary culture of rat retinal microglial cells to gain insight about the mechanistic action of genistein’s anti-inflammatory effect. The results of our experiments indicate that genistein interrupts the glycated albumin signaling cascade in retinal microglial cells that leads to secretion of proinflammatory cytokines. Genistein, when used in 50–100 µM, is a well recognized inhibitor of tyrosine kinase activity in mammalian cells [28]. Thus, its ability to mitigate the glycated albumin-induced TNF-α release indicates the importance of tyrosine kinase activity in mediating this inflammatory response. Consistent with this hypothesis, tyrosine phosphorylation was measured in retinal microglia cells after glycated albumin treatment in the presence/absence of genistein. Microglial recognition of glycated albumin induced an early and significant increase in tyrosine phosphorylation which was reduced by genistein. Because glycated albumin receptors structures and functions remain to be clarified and no consensus tyrosine kinase motif has been identified, the use of genistein, broad-spectrum tyrosine kinase inhibitor, opens the door for future work to characterize which tyrosine kinase is involved after glycated albumin receptor activation.

Besides genistein’s ability to inhibit tyrosine kinase activity, other reported effects might have contributed to our finding of genistein-anti-inflammatory effect, such as possession of antioxidant properties [29]. Emerging evidence indicates that glycated albumin induced NADPH oxidase-dependent ROS formation [30] that might subsequently alter the activity of tyrosine phosphatase which normally antagonize tyrosine kinase activity [31]. With this reduction of antagonistic phosphatase activity, expression of tyrosine kinase activity is unchecked, resulting in accumulation of phosphyorylated tyrosine residues. Therefore, genistein's ability to reduce tyrosine phosphorylation could also be explained in part by blockade of tyrosine phosphatase inactivation via its antioxidant activity. Furthermore, alterations in the activity of transcription factors involved in the inflammation such as NF-κB has been reported to be an important component of NADPH oxidase-dependent redox signaling [32]. NF-κB is a pleiotropic regulator of many pro-inflammatory cytokines that has been found to be activated by a variety of stimuli, including diabetic stress [33,34]. These findings, together with the observations that genistein inhibits NF-κB activation in several cell types under stress conditions [35-37] suggest that the ability of genistein to attenuate glycated albumin-induced TNF-α release could also be explained in part by blockade of NF-κB activation.

Previous studies demonstrated that MAPK activation is necessary for NF-κB activation, induction of target gene expression, and secretion of proinflammatory cytokines from immune cells [38,39]. In agreement, our study identified a signaling cascade in retinal microglia in which glycated albumin binds its receptor, leading to phosphorylation of MAPKs, causing secretion of TNF-α. Furthermore, genistein in our study had an appreciable inhibitory effect on glycated albumin-induced ERK/P38 phosphorylation demonstrating the involvement of tyrosine kinase as a critical upstream signaling event in glycated albumin-mediated ERK and p38 MAPKs activation. Our results are not in agreement with the finding that genistein increases the activity of P38 and inactivates ERK in immortalized human mammary epithelial cells [40]. The cell-specific differences and the type of the stimulator may contribute to these differences in response to genistein. As the ERK pathway is most closely associated with activation of tyrosine kinase receptors [41], blockade of glycated albumin-induced tyrosine phosphorylation by genistein is consistent with its inhibition of ERK activation. Genistein similarly prevented P38 activation by glycated albumin, suggesting that in retinal microglia the glycated albumin receptor signaling cascade directly, or perhaps indirectly, via ROS it generates, leads to p38 phosphorylation.

Collectively, the experiments in this study provide new insights into the mechanisms of the anti-inflammatory effects of genistein, demonstrating that genistein decreases the expression of proinflammatory cytokines in retina of STZ-injected rats and decreases microglial activation, thus affording an anti-inflammatory effect. We also demonstrated that as a tyrosine kinase inhibitor, genistein mitigates glycated albumin-induced TNF-α release from activated microglia through ERK and p38 MAPKs-dependent mechanisms. In conclusion, these data provide preclinical evidence that genistein might be a promising innovative agent in the treatment of the diabetes-induced retinal inflammation. Additionally, our data present new thoughts as to how compounds similar to genistein, which possess tyrosine kinase inhibitory activity, may suppress retinal complications associated with diabetes.
